# Differential features of muscle fiber atrophy in osteoporosis and osteoarthritis

**DOI:** 10.1007/s00198-012-1990-1

**Published:** 2012-04-26

**Authors:** C. Terracciano, M. Celi, D. Lecce, J. Baldi, E. Rastelli, E. Lena, R. Massa, U. Tarantino

**Affiliations:** 1 Department of Neurosciences, Tor Vergata University of Rome, Via Montpellier, 1, 00133 Rome, Italy; 2 Department of Orthopedics and Traumatology, Tor Vergata University of Rome, Rome, Italy; 3 IRCSS Fondazione S. Lucia, Rome, Italy

**Keywords:** Akt, Muscle atrophy, Osteoarthritis, Osteoporosis, Sarcopenia, Type II fibers

## Abstract

**Summary:**

We demonstrated that osteoporosis is associated with a preferential type II muscle fiber atrophy, which correlates with bone mineral density and reduced levels of Akt, a major regulator of muscle mass. In osteoarthritis, muscle atrophy is of lower extent and related to disease duration and severity.

**Introduction:**

Osteoarthritis (OA) and osteoporosis (OP) are associated with loss of muscle bulk and power. In these diseases, morphological studies on muscle tissue are lacking, and the underlying mechanisms of muscle atrophy are not known. The aim of our study was to evaluate the OP- or OA-related muscle atrophy and its correlation with severity of disease. Muscle levels of Akt protein, a component of IGF-1/PI3K/Akt pathway, the main regulator of muscle mass, have been determined.

**Methods:**

We performed muscle biopsy in 15 women with OP and in 15 women with OA (age range, 60–85 years). Muscle fibers were counted, measured, and classified by ATPase reaction. By statistical analysis, fiber-type atrophy was correlated with bone mineral density (BMD) in the OP group and with Harris Hip Score (HHS) and disease duration in the OA group. Akt protein levels were evaluated by Western blot analysis.

**Results:**

Our findings revealed in OP a preferential type II fiber atrophy that inversely correlated with patients’ BMD. In OA, muscle atrophy was of lower extent, homogeneous among fiber types and related to disease duration and HHS. Moreover, in OP muscle, the Akt level was significantly reduced as compared to OA muscles.

**Conclusions:**

This study shows that in OP, there is a preferential and diffuse type II fiber atrophy, proportional to the degree of bone loss, whereas in OA, muscle atrophy is connected to the functional impairment caused by the disease. A reduction of Akt seems to be one of the mechanisms involved in OP-related muscle atrophy.

## Introduction

Osteoporosis, osteoarthritis, and sarcopenia are the most frequent musculo-skeletal disorders affecting older persons. Osteoporosis (OP) is a widespread disorder affecting millions of individuals of all ethnic backgrounds worldwide, particularly among older women [1]. OP is a complex multifactorial disease characterized by reduced bone mass and microarchitectural deterioration of bone tissue, with a consequent increase of fracture risk [2]. Based on a 3-year multicenter survey, the senior author has estimated in Italy an incidence of 410,000 hip, humeral, wrist, ankle, and vertebral fragility fractures. These results confirm that OP is a leading cause of morbidity in the Italian population and a challenging health problem to be addressed by implementing appropriate preventive strategies [3]. There is a rapidly expanding amount of information, based on laboratory studies, indicating that OP is likely to be caused by complex interactions among local and systemic regulators of bone cell function [2]. Osteoarthritis (OA) is a chronic–degenerative joint disease defined by pain, joint stiffness, and a progressive loss of function with considerable impact on the quality of life. OA is one the most frequent causes of disability among the aged, and it is more prevalent in elderly women than in men [4].

OP and OA have been reported in strong association with sarcopenia [5, 6], a term used to indicate the progressive reduction in muscle mass and strength or function that affects older people [7]. Sarcopenia is considered to be one of the major factors responsible for functional limitations and motor dependency in elderly persons [5]. In age-related muscle atrophy, a decrease in both muscle fiber size and number, and a preferential loss of type II fibers, have been reported [8].

Declines in the circulating levels of specific hormones (e.g., estrogens, testosterone, growth hormone, insulin-like growth factor-1 (IGF-1)) have been demonstrated to be associated with sarcopenia and seem to have an important role in its pathogenesis. Similarly, in osteoporotic women, post-menopausal declines in serum hormone levels contribute to increased osteoclastogenesis and bone loss [9, 10].

One of the most important mediators of muscle and bone growth is IGF-1, a peptide hormone, structurally similar to insulin, that exerts its effects through a specific receptor, IGF-1R, that is one of the most potent natural activators of the PI(3)/Akt signaling pathway [10]. Akt acts through different downstream mediators all leading to stimulation of cell growth and proliferation [11].

Sarcopenia in OP and OA is usually evaluated by indirect measures, such as dual-energy X-ray absorptiometry (DXA), bioelectrical impedance, anthropometry, urinary creatine–creatinine ratio, CT or MRI cross-sectional muscle scan, body mass index (BMI), and muscle strength and physical performance tests, whereas direct morphological studies on muscle tissue are lacking [7, 12].

The aim of this study was to analyze, by morphometric analysis, the presence and the degree of muscle atrophy in female patients with OP or OA and evaluate if a correlation between this atrophy and patients’ age, BMI, stage of disease, bone mineral density (BMD) was present. Moreover, we have investigated if the Akt pathway is involved in OP-related muscle atrophy by detecting levels of Akt protein in muscle homogenates.

## Patients and methods

### Subjects

We performed a vastus lateralis muscle biopsy in 15 women with OP undergoing surgery for fragility hip fracture and in 15 age-matched women (age range, 60–85 years) undergoing arthroplasty for hip osteoarthritis. The patients were informed about the experimental procedures and signed an informed consent form before participating in the study. The study was approved by the Ethical Committee of Tor Vergata University Hospital (protocol number 120/06).

### Bone mineral density evaluation

DXA was performed with a Lunar iDXA apparatus (GE Healthcare, Madison, WI, USA). Lumbar spine (L1–L4) and femoral (neck and total) scans were performed, and BMD was analyzed as previously described [13]. Dual-energy X-ray absorptiometry measures BMD (in grams per square centimeter) with a coefficient of variation of 0.7 %. In the OA group, all measurements were performed on the non-dominant side, while participants lay supine on an examination table with their limbs abducted away from the trunk. For the OP group, BMD was measured on the limb opposite the fracture side. Results are expressed as absolute values and as T-scores. Women with fragility hip fracture, a T-score ≤−2.5 SD, and a negative radiographic framework for hip OA were included in the OP group (BMD femoral neck range values, 0.454–0.645 g/cm^2^). Women with a positive radiogram for hip OA and T-score ≥−2.5 SD were included in the OA group (BMD femoral neck range values, 0.845–1.197 g/cm^2^). Patients with neuromuscular diseases, diabetes mellitus, HBV, HCV, HIV infections, smoke or alcohol dependence, or treated with corticosteroids or hormonal drugs for a period exceeding 1 month were excluded from the study. The Harris Hip Score (HHS) of the affected side was calculated in all OA patients. HHS is a scale used to evaluate the degree of pain and functional impairment of the hip joint; it is based on a total of 100 (possible) points, and higher scores indicate better hip function [14].

No significant differences were found in BMI values between the two groups (BMI mean values: OP, 24.4 kg/m^2^; OA, 23.8 kg/m^2^).

### Morphometric analysis

Muscle biopsies were taken from the upper portion of the vastus lateralis during open surgery for hip arthroplasty or for synthesis with intramedullary nail. This muscle was chosen because it is hardly influenced by the fracture event, and it is a good indicator of systemic muscle atrophy related to the disease. Muscle specimens were frozen in melting isopentane and stored at −80 ° until use. Histological evaluations were performed on transverse cryostat sections (7 μm thick) stained with hematoxylin–eosin, Gomori trichrome, ATPase after preincubation at pH 4.2, NADH-dehydrogenase, and cytochrome c oxidase. The presence of other myopathies was ruled out by routine histopathological survey. Morphometric analysis was performed on serial transverse cryostat sections stained with ATPase after preincubation at pH 4.2 as previously described [15]. Morphometric data were obtained by using a semiautomatic image analysis system (QWin Standard V3, Leica, Cambridge, UK). A minimum of 200 muscle fibers per biopsy have been evaluated, comparing type I and type II fibers for relative prevalence, minimum transverse diameter, and cross-sectional area. We accounted as atrophic fibers with a diameter lower than 30 μm, which is the minimum value of the normal range for women [16, 17].

### Immunoblotting

To evaluate whether Akt is involved in OP-related muscle atrophy, muscle homogenates of six OP patients and six age-matched OA control biopsies were immunoblotted, as recently detailed [18]. In brief, 20 μg of protein was loaded into 4–20 % NuPAGE gels (Invitrogen, Carlsbad, CA) and electrophoretically separated. After electrophoresis, samples were transferred to a nitrocellulose membrane. To prevent non-specific binding of the antibodies, the nitrocellulose membranes were blocked in 3 % BSA. They were then incubated overnight at 4°C with a primary antibody against Akt (Cell Signaling Technology, Boston, MA), diluted 1:50. Blots were developed using the Enhanced Chemiluminescence Western Blotting Substrate (Pierce, Rockford, Illinois) in combination with horseradish peroxidase-conjugated secondary antibody (DAKO, Milano, Italy). Protein loading was evaluated by the actin band, and quantification of the immunoreactivity was performed by densitometric analysis using NIH Image J 1.310 software.

### Statistical analysis

Standard statistical procedures were used to calculate means and standard deviation (SD) of age, BMI, and BMD. The statistical significance of the differences in prevalence of fiber type, predominance of fiber atrophy, and Akt muscle protein levels, between the two groups of patients, was determined by Student’s *t* test. Correlation analysis was performed using the Pearson product–moment correlation test; *p* values lower than 0.05 were considered significant; a negative sign indicates an inverse correlation.

## Results

### Prevalence of fiber types

Routine histological stainings showed absence of inflammation, necrosis, regeneration, fibrosis, or other changes in all biopsies, excluding the presence of other muscular diseases. Morphometric analysis performed on ATPase reaction at pH 4.2 did not show any significant difference in fiber type distribution between the two groups of patients. The percentage of type I fibers in OP and OA was 54.72 and 54.81, respectively; the percentage of type II fibers was 45.28 and 45.19, respectively. The absence of a variability in fiber-type prevalence between OP and OA indicates that any difference in muscle fiber diameter between the two groups of patients cannot be ascribed to variation in fiber-type composition.

### Fiber diameter and incidence of fiber atrophy

**Fig. 1:**
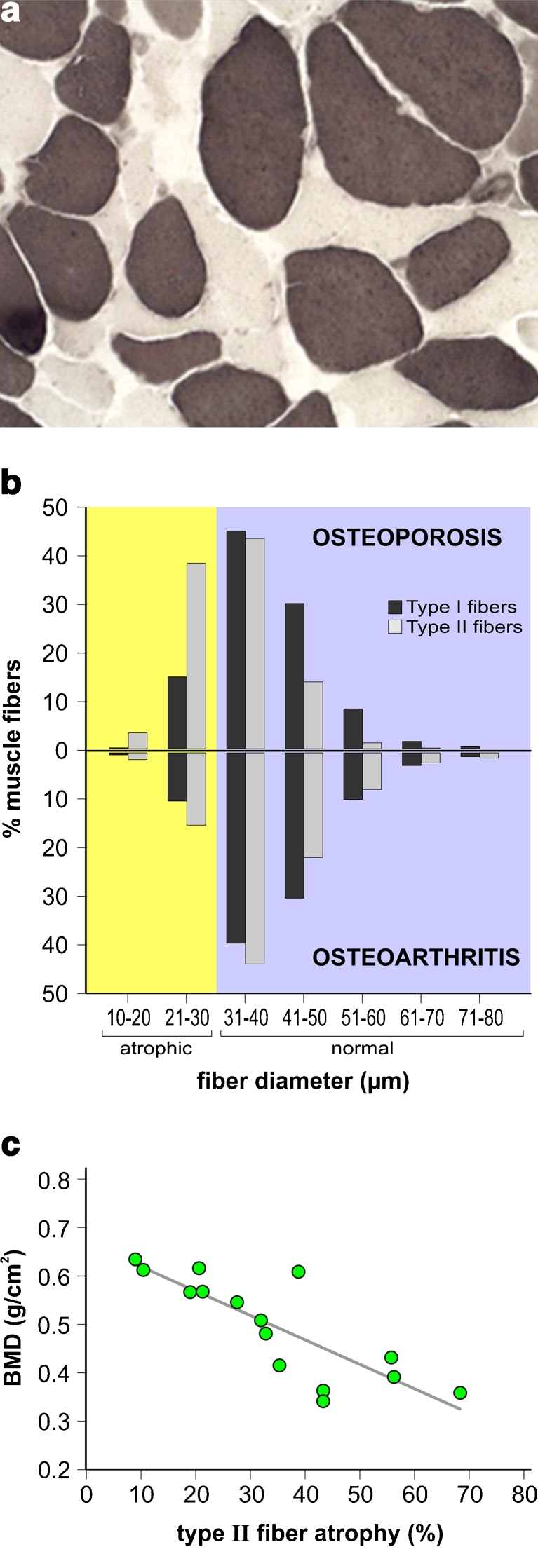
Analysis of muscle fiber atrophy. **a** In osteoporosis, vastus lateralis muscle biopsy reacted for ATPase pH 4.2 shows a preferential type II muscle fiber (*light fibers*) atrophy. **b** Mega-histogram comparing fiber diameter distribution in OP and OA. Type II fibers in the OP group have a higher degree of deviation from the normal distribution toward the atrophic range. **c** Linear regression graph showing in OP an inverse correlation between type II fiber atrophy and BMD

The ATPase reaction showed a diffuse atrophy of type II fibers in the OP muscle biopsies (Fig. 1a). The morphometric analysis of muscle fibers in OP patients showed mean values for minimum transverse diameter ranging between 31.25 and 42.83 μm for type I fibers and between 26.45 and 39.12 μm for type II fibers; mean values for area were ranging from 972.1 to 2,680.2 μm^2^ for type I fibers and between 651.0 and 1,720.3 μm^2^ for type II fibers. In the OA group, the mean fiber diameter was between 35.2 and 50.34 μm for type I fiber and between 33.49 and 53.69 μm for type II fibers; the mean fiber area was between 1,532.8 and 2,792.5 μm^2^ for type I fiber and between 1,644.0 and 2,857.8 μm^2^ for type II fibers.

The analysis of the mega-histogram showed that fiber diameters in the OP group had a higher degree of deviation from the normal distribution toward the atrophic range, compared to OA. This deviation was slight for type I fibers and very prominent for type II fibers (Fig. 1b).

In the OA group, 8.25 % of type I fibers and 12.5 % of type II fibers were atrophic. In the OP group, atrophy was more prominent and involved preferentially type II fibers: in fact, 11.67 % of type I fibers and 36.86 % of type II fibers were atrophic. In both groups, type II fiber atrophy was significantly more frequent than type I fiber (*p* value <0.01), with a threefold ratio in OP and only a 1.5-fold ratio in OA.

On the basis of these raw data, in order to take into account the fact that large deviations from the normal range are more important than small ones, we calculated the atrophy factor (AF) for the different fiber types in both groups, as previously described [15–17]. This analysis showed that the AF for type I fiber was 155 in OP and 110 in OA (normal threshold value, 100). The AF for type II fibers was 451 in OP and 185 in OA (normal threshold value, 200), thus confirming that type II atrophy is a prominent feature in OP only.

### Correlation analysis

To verify if there was a correlation between percentage of muscle atrophy found in these two groups of patients and severity of disease, we performed the Pearson product–moment correlation test. The statistical analysis showed that in OP, the percentage of type II fiber atrophy correlated with neck and total femoral BMD values (correlation coefficient *r* = −0.6 and *p* value <0.05) (Fig. 1c), but not with type I fiber atrophy, patient’s age, and BMI.

In OA, type I and type II fiber atrophy were highly correlated with each other (correlation coefficient *r* = 0.875, *p* value <0.0001) and with disease duration (correlation coefficient *r* = 0.664 and 0.655, respectively; *p* value <0.01), and inversely correlated with HHS (correlation coefficient *r* = −0.730 and −0.562, respectively; *p* value <0.05). No correlation was found between either type I and type II fiber atrophy and patient’s age or BMI.

### Immunoblotting

Considering that muscle homogenates include both normal and atrophic fibers, as well as both type I and type II fibers, we selected OP muscle biopsies showing the higher degrees of type II fiber atrophy, and OA biopsies with the lowest degrees of atrophy, in order to confidently relate the Akt reduction to the preferential type II muscle atrophy found in OP.

**Fig. 2:**
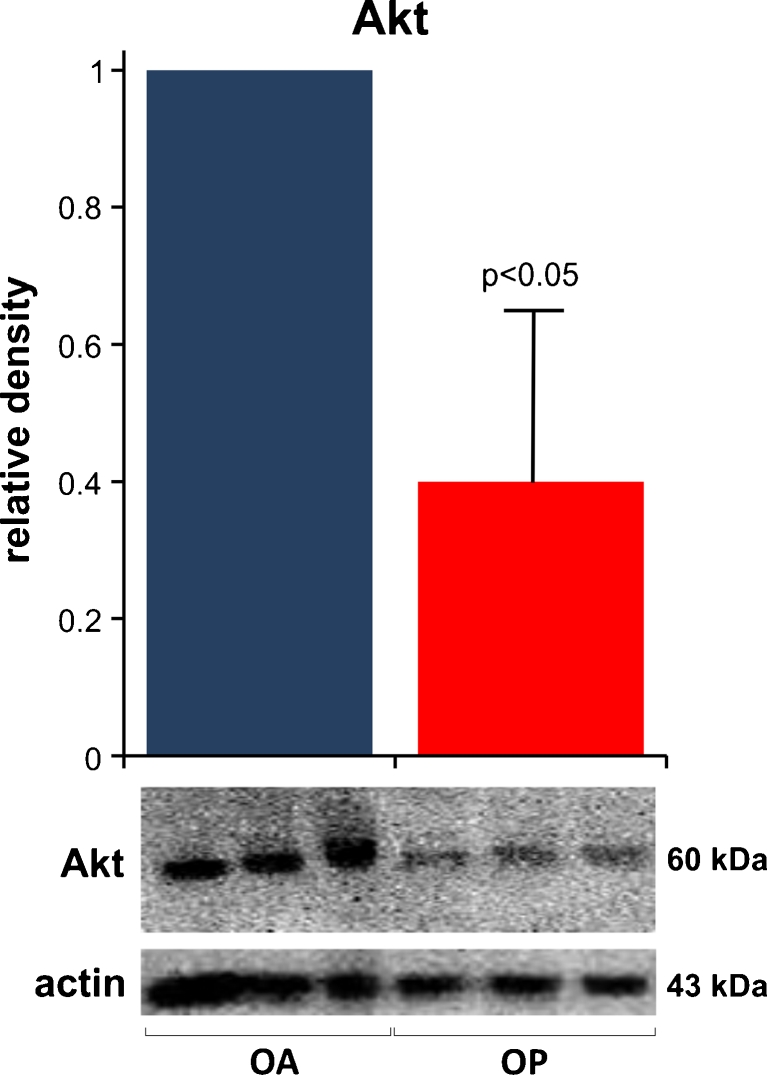
Akt is decreased in OP muscle fibers. Representative immunoblot and densitometric analysis show that in OP muscle, Akt is reduced 2.5-fold as compared to OA. The actin bands indicate protein loading in each sample

To determine whether changes in Akt protein level contribute to the type II fiber atrophy present in OP, we performed immunoblot analysis on six OP muscle biopsies and six OA age-matched control biopsies. In OP muscle, total Akt was decreased 2.5-fold as compared to OA (*p* < 0.05) (Fig. 2).

## Discussion

In this study, we analyzed and compared morphological muscle features associated with OP and OA, the two most frequent skeletal diseases affecting older persons. Those disorders have been both associated to the presence of sarcopenia that, in turn, increases the risk of disability and bone fragility. Our results showed different patterns of muscular involvement in OA and OP. In the latter, muscle atrophy is prominent and affects preferentially type II muscle fibers, with less or no impact observed in type I fibers. This atrophy correlates with BMD, suggesting that disease severity has a central role in the pathogenesis of OP-related muscle atrophy. In OA, muscle atrophy is much less pronounced compared to OP, and is homogeneous among both fiber types. In OA, muscle atrophy is connected with disease duration and patient’s HHS, representing the degree of pain and functional impairment caused by the disease.

A single study has previously reported a higher prevalence of atrophy among type II fibers in osteoporotic patients with low levels of 25-hydroxyvitamin D, although a correlation with the degree of OP was not tested. Unfortunately, many of the biopsies used in that study showed alterations suggestive of concomitant muscular diseases [19].

The OP-related muscle atrophy bears some similarity with other systemic conditions such as cachexia, diabetes, and steroid myopathy, in which a preferential and diffuse involvement of type II fibers has been described [20–22]. In those chronic conditions, a decrease in the levels of specific hormones causes a reduced activation of the IGF-1/PI3K/Akt pathway, the major regulator of postnatal growth of muscle, leading to impaired glucose intake, an altered muscle metabolism, and muscle atrophy. IGF-1 exerts its effects through a specific receptor, IGF-1R, that is one of the most potent natural activators of the PI(3)/Akt signaling pathway. Akt, through different downstream mediators, promotes protein synthesis and retards protein degradation by regulating the expression of various atrogenes [10, 11]. In addition, IGF-1 is able to counteract the effects of myostatin, a member of the TGFβ family involved in muscle atrophy [23]. The hypothesis that Akt is implicated also in OP-related muscle atrophy is supported by our Western blot analysis, showing a significant reduction of Akt levels in OP atrophic muscles, compared to OA muscles. In fact, similarly to other metabolic myopathies, the decline of specific hormones, including IGF-1, occurring in OP might downregulate IGF-1/PI3K/Akt activity, leading to muscle atrophy. Moreover, since IGF-1/PI3K/Akt controls glucose uptake in skeletal muscle [10], its downregulation could affect mainly glycolytic fibers (type II), whereas oxidative fibers (type I) tend to be more resistant to atrophy, because of their capacity of utilizing other substrates than glucose to produce energy. Downstream mediators of Akt, such as mTOR, p70S6K, FoxO1, GSK3b, are to be studied to better clarify the IGF-1/PI3K/Akt role in OP-related muscle atrophy.

An involvement of IGF-1/PI3K/Akt in OP, rather than in OA patients, could explain the muscle morphological differences found in those diseases. In fact, in OP patients, type II fiber atrophy is more prominent and selective as compared to OA and is related to severity of bone mass reduction. All patients in the OP group were examined for the first time on admission because of the hip fracture and did not refer any important limitation in their physical daily activity. This leads to the hypothesis that OP muscle atrophy is independent from muscle disuse. The reduced bone density occurring in OP patients is due mainly to a decrease of circulating hormones, and according to the reduced Akt levels found in OP, we believe that OP muscle atrophy has the same pathogenesis.

Conversely, our OA patients complained of a decline in their physical activity due to pain and functional impairment in the affected joint for some time before surgery, suggesting that their muscle atrophy could be mainly due to disuse. In confirmation of that, OA-related muscle atrophy was of lower extent, more homogeneous among fiber types (even if type II fibers are more liable to size variations), and correlated to the HHS and disease duration. Whether other factors, such as myostatin, systemic or local inflammatory mediators, cytokines, or inflammatory transcription factors, can contribute to the muscle atrophy present in OP and OA should be matter for further investigation.

Our morphological study on vastus lateralis muscles failed to show, in both groups of patients, denervation features such as type grouping or angulated fibers, reported to be present in distal senescent muscle [24] but not in proximal muscle [25]. The lack of correlation between both OP and OA type II fiber atrophy and patients’ age could be explained by the fact that the primary causes of muscle atrophy in these two diseases (hormonal decline and Akt decrease for OP, and disuse due to pain and joint disability for OA) prevail over the effects of physiological muscle aging, as previously described in OA [25].

In summary, muscle atrophy in OP and OA is not related to age and may have different etiologies, the IGF-1/Akt pathway being involved only in OP-related muscle atrophy. Bone mineral density correlated with, and could be used as a marker of, muscle atrophy in osteoporotic patients, whereas disease duration and severity of pain could predict muscle impairment in OA. Further studies need to be performed to better understand the underlying mechanisms of OP- and OA-related muscle atrophy and to ascertain whether similar changes occur also in males.

According to our results, physical activity should be recommended to reduce and prevent OA-related muscle atrophy. Physical activity could be useful also in OP to mitigate muscle atrophy and bone loss due to hormonal decline in the attempt to reduce fracture risk and disability, as previously described [2, 13]. Moreover, pharmacological enhancement of the IGF-1/Akt pathway, to increase protein synthesis and diminish muscle atrophy, might provide a novel therapeutic opportunity in OP-related sarcopenia.
